# Delayed *Plasmodium falciparum* Malaria in Pregnant Patient with Sickle Cell Trait 11 Years after Exposure, Oregon, USA

**DOI:** 10.3201/eid3001.231231

**Published:** 2024-01

**Authors:** Wendi Drummond, Kathleen Rees, Stephen Ladd-Wilson, Kimberly E. Mace, Douglas Blackall, Melissa Sutton

**Affiliations:** Providence Portland Medical Center, Portland, Oregon, USA (W. Drummond);; Washington County Health and Human Services, Hillsboro, Oregon, USA (K. Rees);; Oregon Health Authority, Portland (S. Ladd-Wilson, M. Sutton);; Centers for Disease Control and Prevention, Atlanta, Georgia, USA (K.E. Mace);; Providence Oregon Core Laboratory, Portland (D. Blackall)

**Keywords:** Plasmodium falciparum, parasites, malaria, pregnant patient, pregnancy, sickle cell trait, Anopheles spp., mosquitoes, Oregon, United States

## Abstract

Delayed *Plasmodium falciparum* malaria in immigrants from disease-endemic countries is rare. Such cases pose a challenge for public health because mosquitoborne transmission must be rigorously investigated. We report a case of delayed *P. falciparum* malaria in a pregnant woman with sickle cell trait 11 years after immigration to the United States.

*Plasmodium falciparum* malaria is a major cause of illness and death worldwide (*1*). In disease-hyperendemic areas, most of the population are parasitemic (*2*). Chronic exposure results in partial immunity, and sickle cell trait reduces the severity of infection (*3*,*4*). Delayed *P. falciparum* malaria after immigration to nonendemic countries has been reported in the literature, and pregnancy is the most common risk factor for this unusual presentation (*5*).

Former residents of disease-endemic areas who have *P. falciparum* malaria without recent travel risk present a public health challenge because locally acquired mosquitoborne transmission of the parasite must be ruled out, given the widespread distribution of *Anopheles* spp. mosquito vectors in the United States (*6*–*8*). We report the clinical and public health investigation of a case of delayed *P. falciparum* malaria in a pregnant woman 11 years after immigration to the United States from sub-Saharan Africa.

## The Study

The patient was a 20–30-year-old multiparous pregnant woman from sub-Saharan Africa who came to an emergency department at Providence Portland Medical Center, Portland, Oregon, USA, during her third trimester; she had inadequate prenatal care and a 2-week history of loose stools and abdominal pain before defecation. She reported chills and night sweats without fevers. She denied nausea, vomiting, epigastric pain, runny nose, cough, sore throat, lymphadenopathy, dysuria, or vaginal discharge. The patient was tachycardic; fetal heart rate (FHR) tracing showed a normal FHR, moderate variability, accelerations, and late and variable decelerations. Initial laboratory evaluation on the woman showed microcytic anemia, leukocytopenia, thrombocytopenia, and an increased level of bilirubin (Table).

**Table:** Pregnant patient laboratory values at initial clinical evaluation (day 0) and at discharge (day 5), Washington County, Oregon, USA, July–September 2022

Laboratory test	Day 0	Day 5
Hemoglobin	9.1 g/dL	8.8 g/dL
Hematocrit	27.9 g/dL	27.9 g/dL
Mean corpuscular volume	70.8 fL	72.5 fL
Leukocyte count	3.6 × 10^9^/L	7.3 × 10^9^/L
Platelet count	91.0 × 10^9^/L	102.0 × 10^9^/L
Total bilirubin	2.23 mg/dL	0.92 mg/dL (day 3)

**Figure 1:**
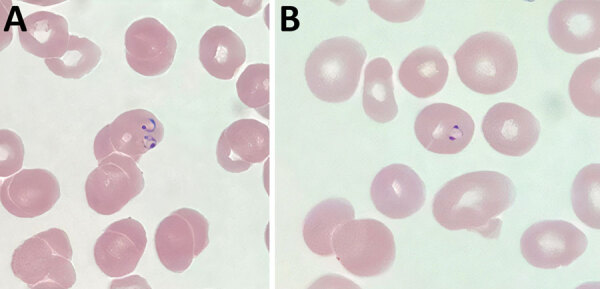
Representative thin blood smears showing *Plasmodium falciparum* in pregnant patient, Washington County, Oregon, USA, July–September 2022. Ring-form trophozoites, morphologically consistent with *P. falciparum*, were identified. A) Multiply-infected erythrocyte showing an applique form; B) ring form showing 2 chromatin dots (headphone form). Original magnification x1,000.

Testing results were negative for HIV, SARS-CoV-2, influenza, hepatitis B, hepatitis C, rubella, and syphilis. The result of a rapid point-of-care BinaxNOW malaria test (Abbott Laboratories, https://www.globalpointofcare.abbott) was positive for *P. falciparum.* Thick and thin malaria blood smears showed *P. falciparum* (Figure 1). Initial parasitemia was 0.2%. We submitted blood smears to the Division of Parasitic Diseases and Malaria diagnostic laboratory, Center for Global Health, Centers for Disease Control and Prevention, and *P. falciparum* morphologic identification was confirmed. A pretreatment blood sample was not available for molecular speciation or whole-genome sequencing.

We initiated a 3-day course of artemether/lumefantrine, and percentage parasitemia decreased to 0.1% within 24 hours. No parasites were observed by day 3 of therapy. The patient received intravenous fluids and 1 unit of packed red blood cells. Maternal tachycardia resolved, and FHR tracing displayed normal FHR with moderate variability, accelerations, and resolution of decelerations. The patient’s anemia and thrombocytopenia improved, and her leukocyte count normalized. The patient gave birth to a healthy postterm infant without evidence of placental insufficiency. Placental pathologic analysis showed sickled maternal erythrocytes, pigment in perivillous fibrin, and mild lymphocytic deciduitis without immunohistochemical evidence of parasites.

The patient immigrated to the United States with her family 11 years before she sought care. She had lived in a metropolitan area of Oregon during the 5 years before she sought care and denied any history of foreign or domestic travel. The patient reported a history of malaria during childhood 19 years earlier, for which treatment was received while living in sub-Saharan Africa. She denied any history of blood transfusions or recent insect bites. The most recent visit to the patient’s home by a person from sub-Saharan Africa occurred 2 years before her illness. She had a history of anemia during previous pregnancies, and her first pregnancy was complicated by thrombocytopenia and preeclampsia. She had a history of sickle cell trait diagnosed by hemoglobin fractionation.

**Figure 2:**
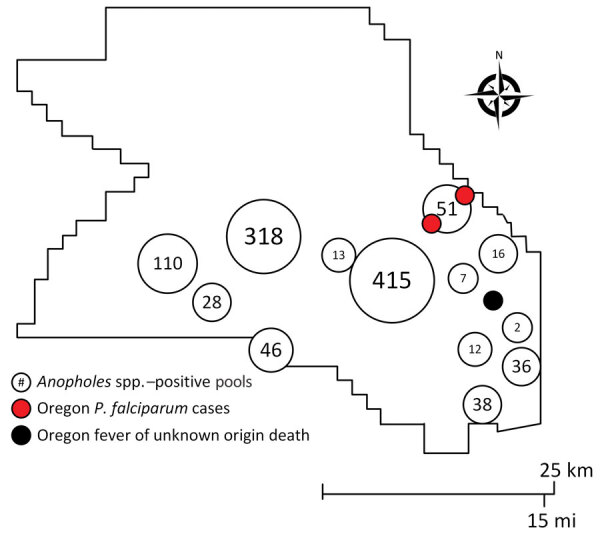
Number of *Anopheles* spp.‒positive pools, *Plasmodium falciparum*‒positive cases, and fever of unknown origin deaths, Washington County, Oregon, USA, July–September 2022.

We explored the plausibility of local malaria transmission by evaluating current mosquito surveillance data and conducting case finding with temporospatial proximity to the case. The investigation was anchored to month of symptom onset (September 2022). We used mapping to visualize spatial associations between mosquito surveillance, malaria case reports, syndromic surveillance, and death surveillance (Figure 2).

*Anopheles freeborni* and *An. punctipennis* mosquitoes were identified during Washington County Public Health’s 2022 Mosquito Control trapping season, May‒September 2022. However, during the period of our investigation, temperatures had decreased, and *Anopheles* mosquitos were not active in the area. *P. falciparum* case finding within the statewide reportable disease database showed 1 travel-associated malaria case with an onset 2 months before this patient and ≈4 miles away. There was no epidemiologic link between the cases; whole blood was not available to identify microsatellite parasite signatures. A search of Oregon’s Electronic Surveillance System for the Early Notification of Community-Based Epidemics for emergency department encounters with a discharge diagnosis of fever of unknown origin (FUO) showed greater than expected activity in the week of the patient’s onset of symptoms (*9*). However, the trend was not isolated to the proximity of the case-patient, and many encounters noted manifestations consistent with viral infections.

An Early Notification of Community-Based Epidemics query for mosquito bites and arboviral diseases did not show greater than expected activity. A vital records query for deaths with an associated diagnosis of FUO showed 1 death temporospatially related to the case. Medical record review by the Malaria Branch, Center for Global Health, Division of Parasitic Diseases and Malaria, Centers for Disease Control and Prevention, ruled out the death as related to malaria due to clinical and laboratory incompatibility. Thus, there was no evidence to support local mosquitoborne transmission.

## Conclusions

We report a case of *P. falciparum* malaria in a pregnant woman 11 years after immigration from sub-Saharan Africa to the United States. To assess for local mosquitoborne transmission, a joint state and local public health investigation examined mosquito surveillance data, performed case finding for additional malaria cases, and reviewed syndromic surveillance and death surveillance for FUO diagnoses with temporospatial proximity to the case. This comprehensive assessment enabled the public health departments to effectively evaluate local mosquitoborne transmission and emerging local risk.

Although delayed *P. falciparum* illness has been documented, it remains rare, and the patient’s latency period was unusually long at 11 years. In 1 case series and literature review, pregnancy was the most prevalent risk factor associated with delayed presentation and reports of delayed presentation in pregnant women ranged from 3 months to 4 years (*5*). Delayed *P. falciparum* in persons from disease-endemic regions is believed to arise from persistent low-level parasitemia and decaying *P. falciparum*-specific immunity (*5*). In pregnant women, pregnancy-related immunosuppression, sequestration of *P. falciparum* parasites in the placenta, and, possibly, placental antigen expression might increase the risk for delayed *P. falciparum* presentation (*10*–*12*). The patient’s sickle cell trait might have also contributed to the latency of her delayed presentation. Sickle cell trait protects against severe disease from *P. falciparum* infection and is associated with lower parasite densities and delayed malaria (*13*,*14*).

Most malaria cases in the United States are related to travel to a disease-endemic region. However, malaria can rarely be acquired locally through mosquito bite, transfusion, or other parenteral route, transplantation, or during pregnancy or childbirth (*15*). Our case-patient had no known history of transfusion or transplantation, and her infant did not show development of malaria. Although the patient denied traveling to a disease-endemic area, she was not available for follow-up, and we were unable to verify travel history through a passport review. Therefore, undisclosed travel to a malaria-endemic country remains an unlikely possibility.

Malaria should be considered in all patients from disease-endemic regions who have compatible symptoms regardless of time since exposure. Clinical suspicion should be heightened in persons who have underlying risk factors for delayed manifestation, including pregnancy, immunosuppression, and sickle cell trait. To rule out the possibility that a patient without recent travel risk acquired malaria locally, rigorous public health investigation is required. Components of an investigation might include medical and travel record review, environmental surveillance, case finding, and syndromic and death surveillance with consideration of temporospatial proximity to the case.
